# Supplementary material for: X-ray rheography uncovers planar granular flows despite non-planar walls
Source: Nat Commun. 2018 Nov 30;9:5119. doi: 10.1038/s41467-018-07628-6 (PMC6269474; doi:10.1038/s41467-018-07628-6)
Supplement: Supplementary file 1 — Supplementary Information [file 41467_2018_7628_MOESM1_ESM.pdf]

Supplementary Information for
"X-ray rheography uncovers planar granular flows despite non-planar walls"

James Baker[1], François Guillard[1], Benjy Marks[1] & Itai Einav[1*]

[1] *School of Civil Engineering, The University of Sydney, NSW 2006, Australia*

[*] *Email: itai.einav.sydney.edu.au*

Supplementary methods: validation using analytical velocity field

The rheography technique is validated in this supplementary methods section by generating sets of artificial radiographs, or forward projections, with particle positions being updated at each timestep according to prescribed motions. The reconstruction process described in the main text is then applied to these images to recover the underlying velocity fields.

We enforce a continuous steady-state flow, where an individual particle's velocity is explicitly determined from its spatial position alone by an analytical expression. This allows precise calculation of the errors introduced at each stage of the reconstruction process. For simplicity we use a unidirectional flow, which requires only two perpendicular projection angles, but the same principals can be applied to fully three-dimensional cases. However, such analytical flow fields do not necessarily follow physical packing constraints, and thus directly translating the results of this validation technique to real flows is difficult. The alternative validation method, which is presented in the main text and involves generating radiographs from realistic DEM simulations, overcomes this issue, but is less appropriate for precisely quantifying errors.

The generation of artificial radiographs requires a model for how X-rays travel through the medium, which is taken to be the Beer-Lambert exponential attenuation law[1],

$$I(\mathbf{x}) = I_0 \exp\left(-\int_l (\mu/\rho)\rho_b(l)\,\mathrm{d}l\right). \tag{1}$$

This describes, at a planar detector position $\mathbf{x}$, the final intensity I of an incident ray of strength I_0 as it travels along a path l, where μ is the attenuation coefficient, ρ the material density and

ρ_b the bulk density. We consider an idealised system where the sample is composed of either solid glass particles, each of the same uniform material density, or interstitial air. Since the X-ray attenuation coefficient in air is several orders of magnitude lower than in glass, these contributions are negligible compared to the solid phase. If we also assume a parallel X-ray beam with no scattering then the expression (1) can be simplified to

$$I(\mathbf{x}) = I_0 \exp\left(-\mu D(\mathbf{x})\right), \tag{2}$$

where μ is the constant attenuation coefficient and $D(\mathbf{x})$ represents the integrated thickness of solid material that a ray at in-plane position $\mathbf{x}$ travels through.

Using expression (2), initial radiographs are generated by assuming that 4000 spherical particles of mean diameter $20\ \text{px} \pm 10\%$ are positioned at random locations inside a domain with dimensions $512\ \text{px} \times 1024\ \text{px} \times 1024\ \text{px}$ in the x, y and z directions respectively. 8-bit images representing projections through the y and z directions are then computed by directly taking the theoretical intensity (2) as the grayscale value, with parameter $I_0 = 1$ and $\mu = 0.005\ \text{px}^{-1}$, as shown on Supplementary Figure 1.

Now, at each time step the particle positions are updated, and new radiographs are generated, by assuming that grains move according to the unidirectional velocity field $\mathbf{u} = (u, 0, 0)$, where

$$u(y, z) = u_0 y(1 - y)z. \tag{3}$$

The units in (3) are assumed to be pixels per time step, with the normalised coordinates $y, z \in [0, 1]$ and u_0 a constant magnitude. Such a flow field bears superficial resemblance to steady uniform

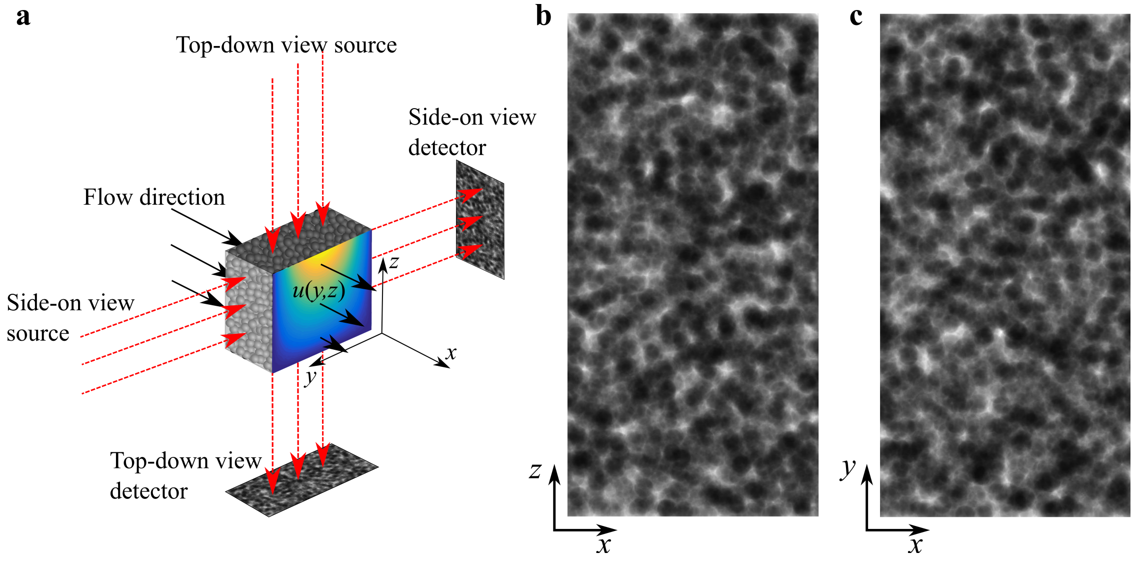

Supplementary Figure 1. Analytical velocity field setup. Panel (a) shows the orientation of the two different projection angles, with the artificial X-ray beams shown with red arrows and the unidirectional velocity field, $u(y, z)$ from equation (3), shown with black arrows and coloured plane. Panels (b) and (c) show the resulting artificial radiographs for the side-on and top-down views, respectively, with grayscale values determined by expression (2). Supplementary movie 2, showing the evolving radiographs at each timestep, is available online.

chute flow[2], where motion is primarily in the downslope direction and is independent of the x-coordinate. The domain is taken to be periodic in the x direction.

Deconvolution process errors. The first step in the velocity reconstruction process is obtaining the velocity probability density functions (PDFs) in each interrogation window. This requires splitting the radiographs into discrete windows and computing the auto-correlation (A) and cross-correlation (C) functions. Note that, due to the one-dimensional displacement field, the definitions

are altered slightly and become

$$A(m) = \sum_p \sum_q \frac{(I_1(m+p,q) - \bar{I}_1)(I_1(p,q) - \bar{I}_1)}{\sigma_1^2}, \tag{4}$$

$$C(m) = \sum_p \sum_q \frac{(I_1(m+p,q) - \bar{I}_1)(I_2(p,q) - \bar{I}_2)}{\sigma_1 \sigma_2}. \tag{5}$$

for successive images I_1 and I_2. Specifically, a two-dimensional patch is still used but we are only seeking one-dimensional displacements, meaning the computed A and C are already one-dimensional functions. These correlation functions are then averaged over many time steps and are used to directly compute the one-dimensional PDFs by solving the deconvolution inverse problem, given by equations (4)–(6) in the main text. Finally, these deconvolutions are averaged over the x-direction to give a single PDF for each distinct z position (for side-on view radiographs) or y position (for top-down view radiographs).

To calculate the errors introduced during this deconvolution process, the analytical velocity PDFs are also computed for each interrogation window. We then calculate the normalised Euclidean distances between the exact and reconstructed PDFs at all spatial positions, before averaging over all windows to give a single mean error for each computation. The results are shown on Supplementary Figure 2, where it can be seen that increasing the number of correlation-function averages generally reduces the overall error, albeit in a non-monotonic fashion. Supplementary Figure 2a shows how the size of the interrogation window influences the mean error, with larger boxes incorporating more particles and thus improving the statistical measure of velocity. However, using very large window sizes will reduce the spatial resolution of the final solution, meaning the optimal size will be a trade-off between these competing factors. The effect of the velocity

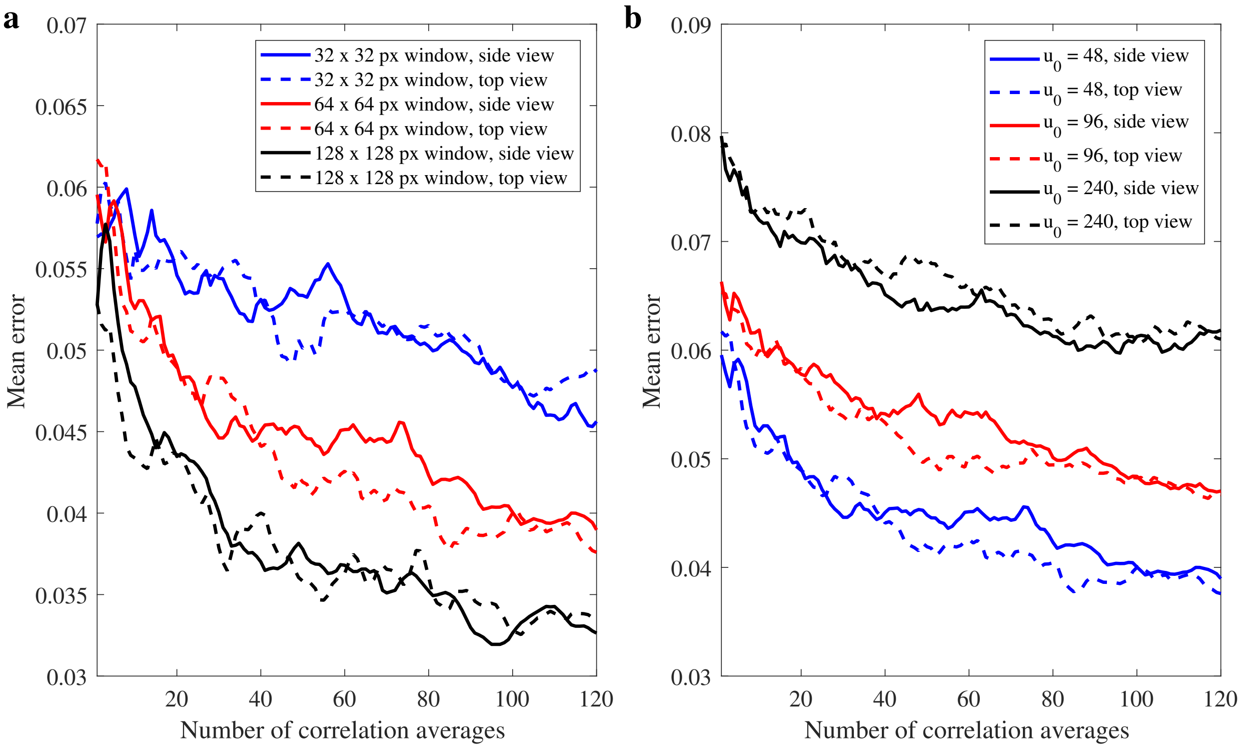

Supplementary Figure 2. Deconvolution process errors. Plots of the mean error between analytical velocity PDFs and those obtained through the deconvolution process, as a function of the number of correlation averages. Panel (a) shows the effect of changing the size of the interrogation window for a fixed velocity magnitude $u_0 = 48$ (from equation (3)). Panel (b) shows the influence of u_0 for a fixed window size of 64×64 px. In both cases solid lines denote side-on view projections (beam in y-direction) and dashed lines are the top-down view (z-direction beam path).

magnitude, u_0 in equation (3), is displayed on Supplementary Figure 2b. Here we see that larger displacements lead to less accurate deconvolutions, because it becomes more difficult to correlate individual particles between images. Nevertheless, in all cases the errors remain less than 10% and therefore within acceptable bounds.

Discretisation process errors. The next stage in the reconstruction process is the discretisation of the velocity PDFs into 'candidate' vectors by splitting into equally-spaced percentiles. Intuitively, the errors between these candidate vectors and the exact underlying velocity field must tend to zero as the discretisation gets successively finer. This is because the PDFs can be thought of as the large N limit of evaluating the velocity at N evenly distributed spatial positions, and then binning to the desired level of precision. Our discretisation process is the exact inverse of this process when the PDF is split up into the same number of points (N) that were used to form it.

In practise, errors are introduced because the velocity PDFs are discretised into significantly fewer vectors. To investigate the effect of such data coarsening, for each viewing direction and interrogation window the analytical PDFs are split into the desired number of candidate vectors. The corresponding exact velocity vectors are then calculated as the expected values over the corresponding internal voxels. These are sorted into numerical order, allowing the error between the exact and candidate vectors to be calculated for each sorted index. These errors are then averaged over all indices, giving a single mean error for each window. Supplementary Figure 3 shows the results for all windows, with the top-view (Supplementary Figure 3a) giving the mean errors at different cross-slope (y) positions and the side-view (Supplementary Figure 3b) at different heights

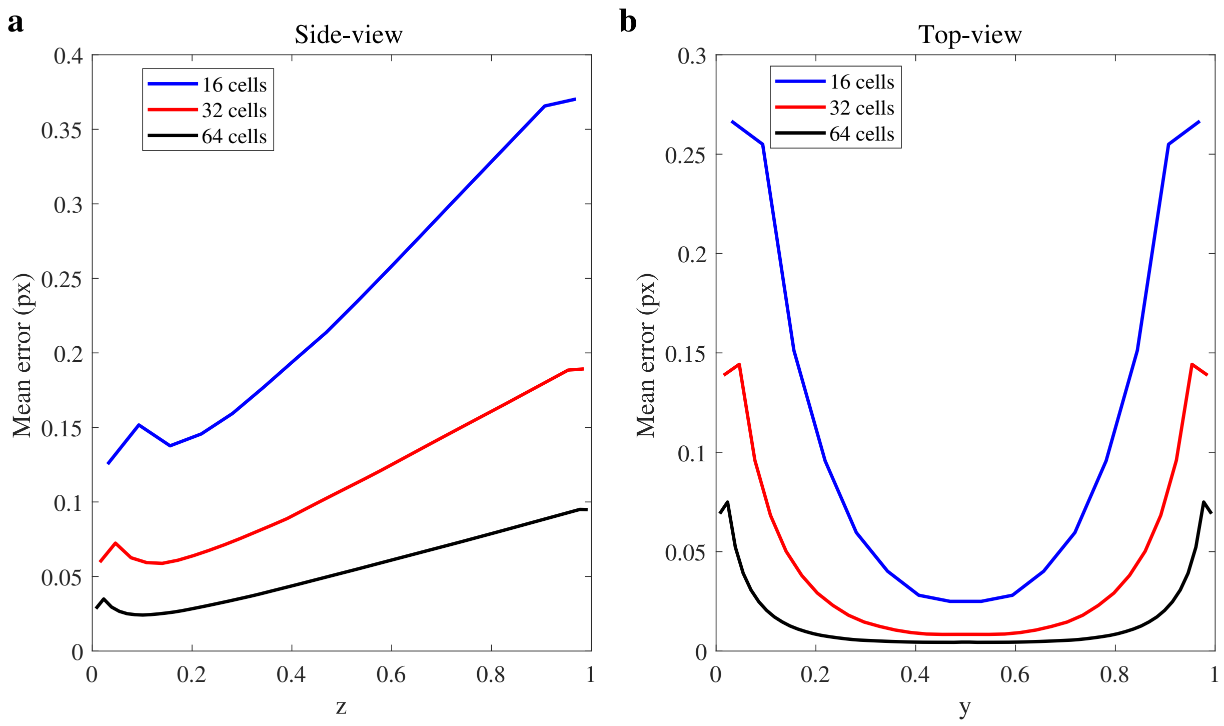

Supplementary Figure 3. Discretisation process errors. Plots of the mean error between the 'candidate' vectors (obtained by discretising exact velocity PDFs with $u_0 = 48$) and analytical velocity field vectors, as a function of spatial position. Panel (a) shows the results when imaging from the side, with errors calculated at different heights z, and panel (b) shows the results from the top (errors at different cross-slope positions y). Different coloured lines correspond to different numbers of discretisation cells.

z. Both cases show that the additional errors introduced at this stage in the process are very small, being less than 0.4 pixels, or 1% of the mean flow velocity. As expected, increasing the number of discretisation cells improves the overall accuracy, with the error appearing to be inversely proportional to the cell count. Interestingly, for the side-view results the errors are largest towards the top of the flow, where particles are travelling faster, whereas the top-view results show more significant errors near the slow-moving boundaries. This suggests a complex relationship between velocity magnitudes, gradients and interrogation window size that could warrant further investigation.

Matching process errors. The final stage involves taking the two sets of candidate arrays, from the side view and top view, and using them to reconstruct the full internal field in a single (y, z) slice. We refer to this as a Sudoku-style problem due to the parallels with such puzzles. The approach is based around minimising the 'matching error' between the two sets of observations, given by equations (8) and (9) in the main text methods. We do not carry out the full optimisation procedure, instead calculating the matching error for many different paths and selecting those paths with the smallest discrepancies to average for the final solution. There are therefore two important parameters in this matching process: the total number of solution iterations computed and the number kept for the averaging process.

Supplementary Figure 4 investigates the effect of the total number of solution iterations, showing how the mean error between the exact and reconstructed velocity fields decreases with increasing number of iterations. In all cases the single path with the smallest matching error is chosen for the reconstruction. The reconstruction is carried out using two types of candidate arrays, firstly using the exact ordered velocity vectors from the analytical solution (Supplementary Figure 4a) and secondly using those obtained from discretising the PDFs (Supplementary Figure 4b). We see that the final errors are significantly lower for the former, which is to be expected since it is possible to achieve perfect matching between vectors from different directions. However, even when using the discretised PDFs, where perfect matching is no-longer likely, the mean errors remain small. Supplementary Figure 4 also shows how the required spatial resolution of the internal reconstruction influences the total error. Perhaps counter-intuitively, reconstructing velocities on a finer grid improves overall accuracy. This could be attributed to the fact that each individual vector

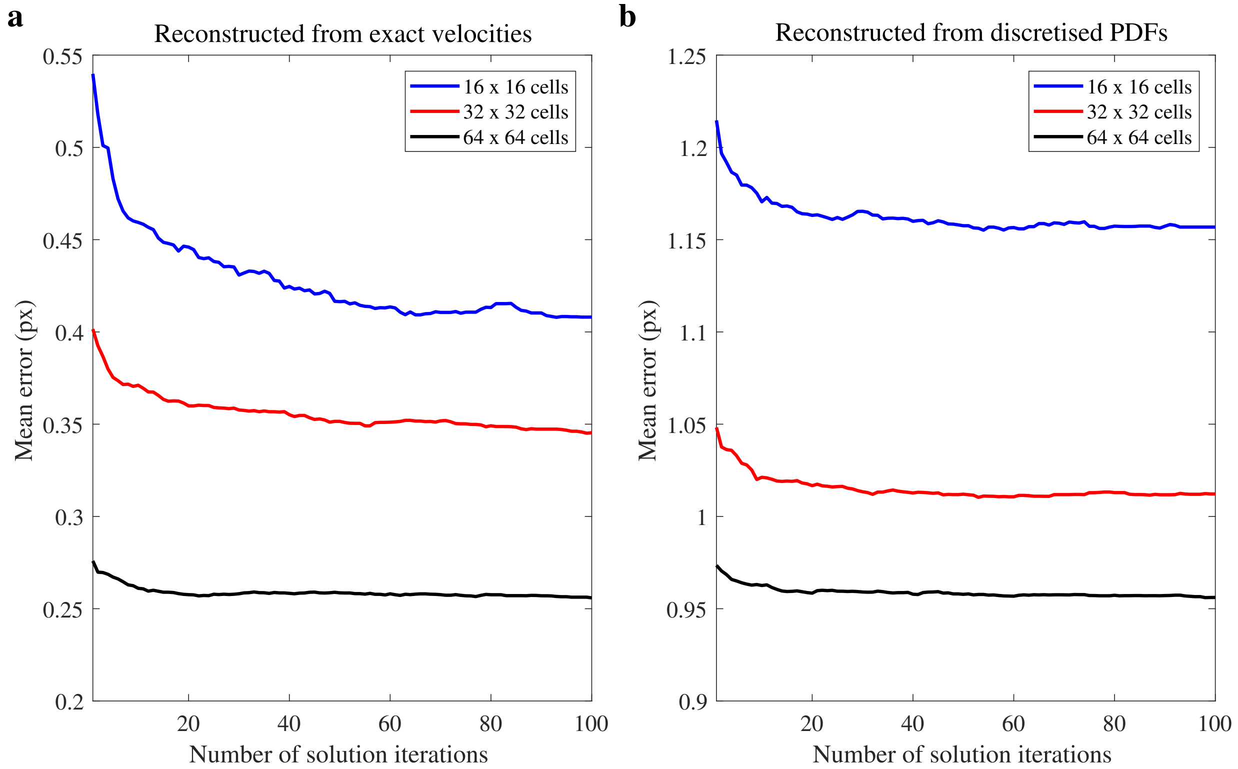

Supplementary Figure 4. Effect of solution iterations on matching process errors. Plots of the mean error between the exact velocity field and the velocity field reconstructed from the final matching process of candidate arrays, as a function of total number of solution iterations. In all cases the single iteration with the smallest matching error is selected as the final solution. Panel (a) shows results using the sorted exact velocities as the candidate arrays, whereas panel (b) uses the candidate arrays obtained by discretising the velocity PDFs. Different colours represent different spatial resolutions for the internal reconstruction.

represents a smaller proportion of the overall map, meaning the matching process is more forgiving if a single cell is sub-optimally assigned. Of course, such higher spatial resolutions require increased computational power at all stages in the reconstruction process.

The second important reconstruction parameter, the number of solution averages, is investigated on Supplementary Figure 5. Here, 1000 different solution iterations are calculated in all cases and the final answer is obtained by averaging over different numbers of lowest-matching-error paths. Supplementary Figure 5 shows the mean errors between the analytical and reconstructed solutions, which typically decay as larger numbers of solutions are taken into account. However, if one continues to increase the number of solutions whilst keeping the total number of iterations constant, those paths with high matching errors start to play a role in the final answer. Because these are less accurate solutions, they will have a negative effect on the accuracy of the average velocity field. For this reason, the mean error may saturate, or even increase, with large numbers of solutions averages, which is particularly evident for the 16×16 cell results on Supplementary Figure 5. Choosing the optimum number of solutions to keep in the final answer will depend on the total number of computed iterations since, if more paths are calculated in total, more are likely to be highly accurate.

Overall process errors. The above subsections discuss the errors introduced in the three main steps of the reconstruction process. For the case considered here, the largest sources appear to be in the deconvolution process and, to a lesser extent, in the final matching process. The discretisation of the velocity PDFs into candidate arrays only introduces very small errors, providing the mesh is

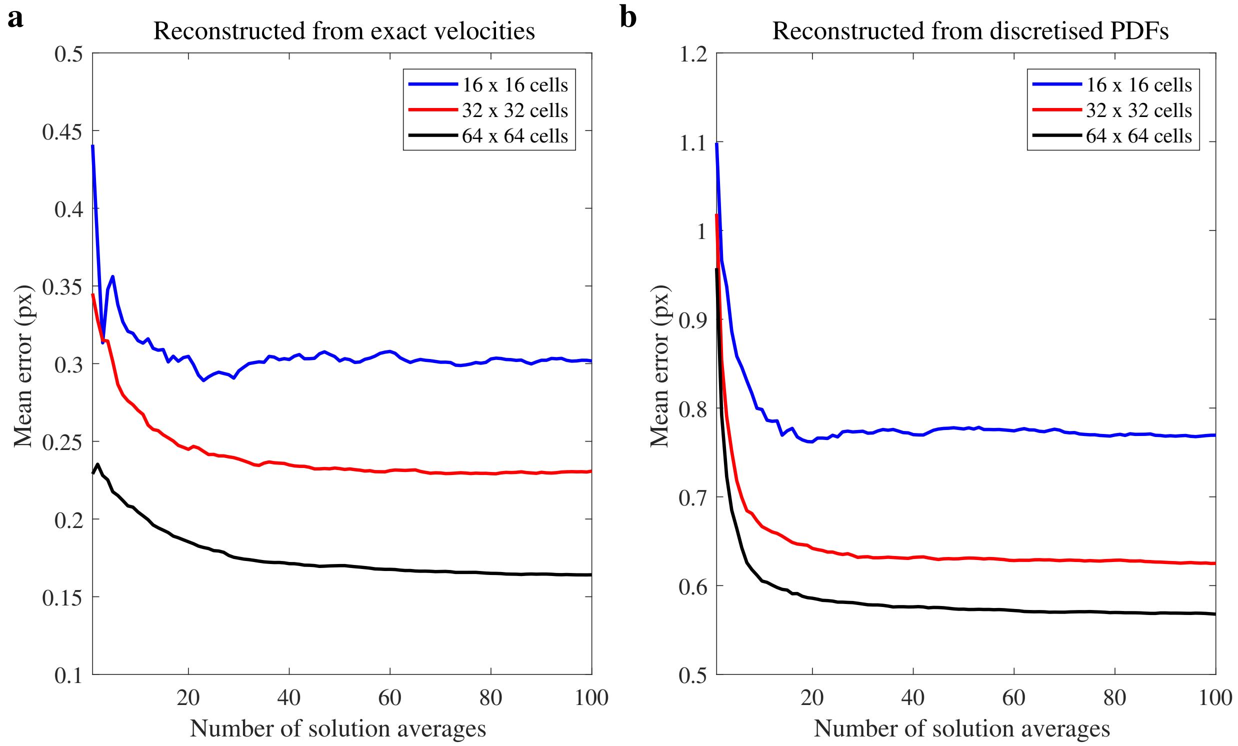

Supplementary Figure 5. Effect of solution averaging on matching process errors. Plots of the mean error between the exact and reconstructed velocity field, as a function of number of solution averages. In all cases 1000 total solution iterations are computed, and the required number with the smallest matching errors are averaged to give the answer. Panel (a) shows results using the sorted exact velocities as the candidate arrays, whereas panel (b) uses the candidate arrays obtained by discretising the velocity PDFs. Different colours represent different spatial resolutions for the internal reconstruction.

fine enough. The relative errors for other flow regimes and parameters may differ, but these results should at least provide useful guidelines.

The next question to be addressed is whether the accumulation of the different errors has a detrimental effect on the final reconstruction. To this effect, the whole process has been followed from artificial radiograph generation to matching of candidate arrays, and Supplementary Figure 6 shows the final results. It can be seen that the comparison to the analytical velocity is generally very good, with mean errors being calculated as less than 15%. There does, however, appear to be some smearing of velocity gradients in the reconstructed flow field, which may be a result of the enforced regularisation in the deconvolution process, as well as the path-averaging approach.

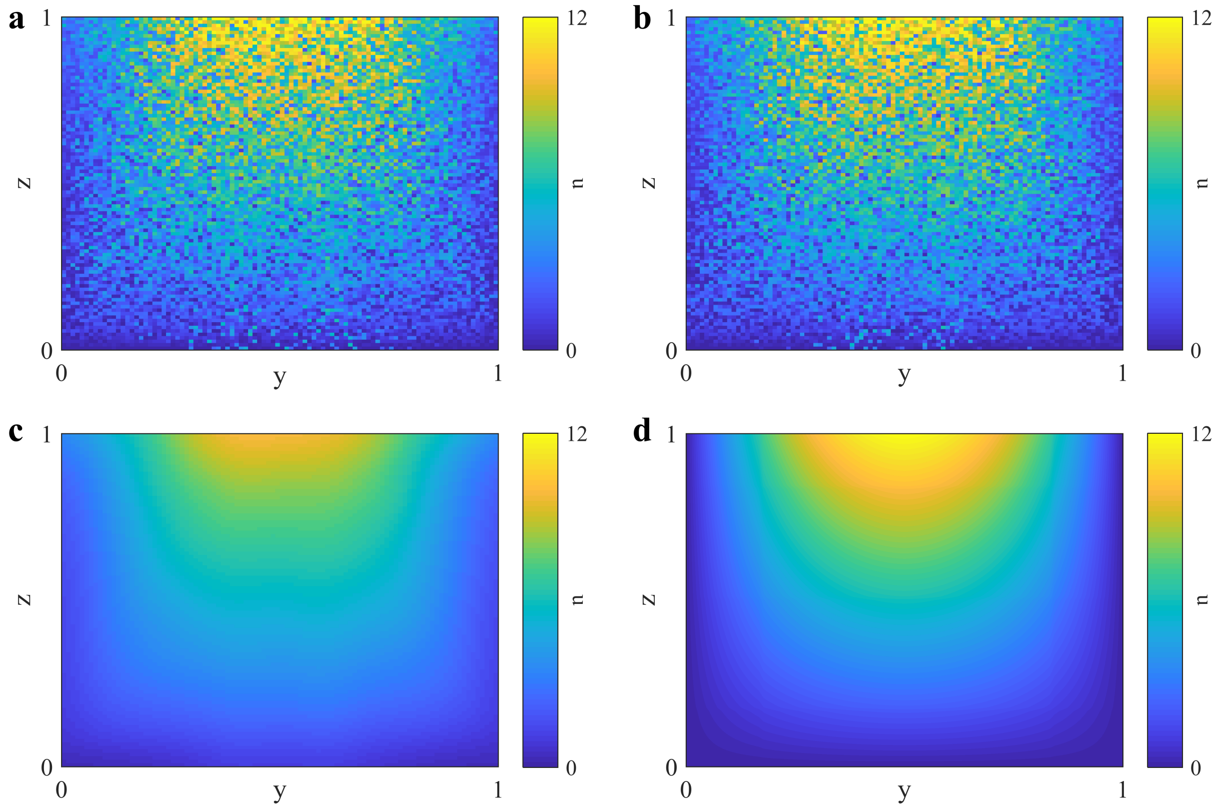

Supplementary Figure 6. Comparison between rheography results and exact velocity field. Panels (a) and (b) show two realisations of the reconstructed velocity field for two distinct paths, and panel c shows the mean of 100 such paths. For reference, exact analytical velocity, equation (3) with magnitude $u_0 = 48$, is shown on panel d. Reconstructions show the result of the complete process, beginning with correlation analysis of the artificial radiographs.

Supplementary References

1. Krinitzsky, E. L. *Radiography in the Earth Sciences and Soil Mechanics* (Springer, 1970).

2. Jop, P., Forterre, Y. & Pouliquen, O. Crucial role of sidewalls in granular surface flows: consequences for the rheology. *J. Fluid Mech.* **541**, 167–192 (2005).
