# Supplementary material for: X-ray rheography uncovers planar granular flows despite non-planar walls
Source: Nat Commun. 2018 Nov 30;9:5119. doi: 10.1038/s41467-018-07628-6 (PMC6269474; doi:10.1038/s41467-018-07628-6)
Supplement: Supplementary file 3 — Description of Additional Supplementary Files [file 41467_2018_7628_MOESM3_ESM.pdf]

Description of Additional Supplementary Files

Supplementary Movie 1

Experimental and artificial radiographs from three orthogonal projection angles. Movie showing successive radiographs of the experimental geometry acquired from three orthogonal projection angles at 30 fps. Top images are artificially generated using discrete element method simulations, middle images are real radiographs from experiments using glass spheres, and bottom images are real radiographs from pearl barley experiments.

Supplementary Movie 2

Artificial radiographs for analytical velocity field. Movie showing artificial radiographs generated by assuming an analytical,unidirectional flow field, as described in the Supplementary methods. Left panel represents side-on projections, whereas right panel represents top-down projections.
